# Results of the implementation of a double-check protocol with point-of-care ultrasound for acute heart failure in the emergency department

**DOI:** 10.1186/s13089-024-00373-6

**Published:** 2024-04-17

**Authors:** Tomás Villén, Yale Tung, Rafael Llamas, Fernando Neria, César Carballo, José Luis Vázquez, Diana Monge

**Affiliations:** 1 School of Medicine, Universidad Francisco de Vitoria, Madrid, Spain; 2 Internal Medicine Department, Hospital Universitario La Paz, Madrid, Spain; 3 Emergency Department, Hospital Universitario Reina Sofía, Córdoba, Spain; 4 Emergency Department, Hospital Universitario Ramón y Cajal, Madrid, Spain; 5 Pediatric Intensive Care Unit, Hospital Universitario Ramón y Cajal, Madrid, Spain

**Keywords:** Ultrasonography, Heart failure, Diagnosis, Diagnostic errors

## Abstract

**Objective:**

To determine the effectiveness of a double-check protocol using Point-of-Care Ultrasound in the management of patients diagnosed with Acute Heart Failure in an Emergency Department.

**Method:**

Prospective analytical cross-sectional observational study with patients diagnosed with Acute Heart Failure by the outgoing medical team, who undergo multi-organ ultrasound evaluation including cardiac, pulmonary, and inferior vena cava ultrasound.

**Results:**

96 patients were included. An alternative diagnosis was found in 33% of them. Among the 77% where AHF diagnosis was confirmed, 73.4% had an underlying cause or condition not previously known (Left Ventricular Ejection Fraction less than 40% or moderate-severe valvulopathy). The introduction of the protocol had a clinically relevant impact on 47% of all included patients.

**Conclusions:**

The implementation of a double-check protocol using POCUS, including cardiac, pulmonary, and inferior vena cava assessment in patients diagnosed with Acute Heart Failure, demonstrates a high utility in ensuring accurate diagnosis and proper classification of these patients.

## Background

Emergency Departments (ED) constitute one of the cornerstones of virtually any healthcare system when it comes to managing a high volume of patients and are also the source of most hospitalized patients, facing a very significant number of diverse pathologies on a daily basis, many of which are time-dependent and require a highly accurate diagnosis in a short time. Among them, Acute Heart Failure (AHF) is particularly prevalent, accounting for a high percentage of all ED visits due to shortness of breath or dyspnea [1], and also requiring an accurate and early diagnosis upon which its morbidity and mortality depend. To achieve this, Emergency Physicians have a diagnostic arsenal which, although it has been developing and increasing in recent years, is still not fully accurate. On one hand, the scarcity of time and the convergence of multiple patients simultaneously, combined with the limited accuracy of traditional medical history and physical examination, and the imprecision of standard complementary tests (ECG and chest X-ray) do not provide an ideal starting point. The emergence of biomarkers, especially NT-proBNP, has significantly improved the diagnosis, but its specificity for AHF diagnosis is also not optimal since it also elevates in multiple clinical scenarios. All of this makes the possibility of a misclassification of AHF patients in ED a very high percentage [2].

Furthermore, adding Clinical Ultrasound (CU) presents the advantage of being performed at the patient’s bedside, evaluating multi-organ function with the mission of revealing real-time pathophysiology underlying a patient whose symptoms or signs suggest AHF. Although the individual assessment of the heart, lungs, and inferior vena cava is fairly accurate, when performed collectively, the accuracy is optimized [3]. The aim of this study is to understand the impact that a dual-check protocol with CU has on patients who have been diagnosed with AHF through a “traditional” approach (medical history, examination, complementary tests, and biomarkers) in terms of detecting misclassified patients and the additional information revealed by CU that is relevant from a diagnostic and therapeutic standpoint.

## Methods

This is a prospective analytical cross-sectional observational study conducted at a tertiary-level hospital. Following approval from the Ethics and Research Committee, adult patients were included who appeared during the morning shift rounds with a diagnosis of AHF made without POCUS using opportunistic sampling (when the expert, not part of the treating team, performing POCUS was present) and obtaining informed consent. Exclusion criteria included hemodynamic instability or cardiogenic shock, patient refusal, or AHF not being considered the primary diagnosis for their Emergency Department stay by the outgoing treating team.

After the ultrasound assessment, the evaluator communicated the findings to the treating team, discussing the images obtained, and the course of action was decided accordingly taking in consideration the whole clinical picture (ultrasound, clinical information, other tests). In order to ensure the accuracy of the diagnosis made by the protocol, the final diagnosis in the clinical ward (when the patient was eventually admitted) when available.

### Ultrasound evaluation

The study evaluators were certified Emergency Medicine experts by national and international scientific societies with over 10 years of experience. After collecting demographic parameters, a systematic evaluation was carried out using an ultrasound machine, including cardiac ultrasound with a phased-array probe, lung ultrasound, and ultrasound evaluation of the inferior vena cava using a curvilinear probe. Among the ultrasound parameters, left ventricular ejection fraction (LVEF) was measured using a biplane Simpson technique, categorized as < 40% or > 41%. Diastolic pattern was assessed using pulsed Doppler flow across the mitral valve and Tissue Doppler at the mitral annulus level. Valvular pathology was evaluated using continuous wave Doppler. For lung evaluation, a technique involving the division of the chest into 8 zones [4] was performed (anterior and laterals) using curvilinear probe and lung preset. The number of B-lines was counted using automatic calculation (auto B-lines) [5]. Similarly, for the assessment of the inferior vena cava, the automatic calculation tool for diameters and collapsibility percentage (auto IVC) was used, given its excellent correlation with expert visualization [6]. The number of B-lines per area (0 to 5) was used to calculate a lung score, [7] the arithmetic sum of these lines, ranging from 0 to 40, as a measure of lung aeration. Regarding the pulmonary diagnosis, a pneumonia was considered when a consolidation pattern or a focal B lines pattern were visualized, and respiratory infection with several and/or bilateral foci of B lines with subpleural consolidations appearances. Although the performance time was not originally recorded, evaluators indicated that entire protocol was performed in less than 15 min at the patient’s bedside.

### Statistical analysis

Qualitative data were presented as absolute frequencies (n) and relative percentages (%), while quantitative data were presented as mean ± SD or as median [Interquartile Range] depending on the normality of distribution. Comparison between patients with and without an alternative diagnosis was performed with Wilcoxon rank sum test, Pearson’s Chi-squared test or Fisher’s exact test. Significance was considered when the p-value obtained was less than 0.05. The “cutpointr” package in R was used to obtain ROC curves and determine optimal cutoff points for maximum and minimum diameters and collapsibility coefficient of the inferior vena cava [8]. The optimal cutoff point was adjusted using the Youden Index, and diagnostic test parameters (Sensitivity, Specificity, ppv - Positive Predictive Value, and npv - Negative Predictive Value) were calculated. The decision tree for alternative diagnoses was obtained using the “rpart” and “rpart.plot” packages, with 80% of the samples used to determine the decision tree and the remaining 20% to validate it.

All analyses were performed using R software version 4.2.3.

## Results

There were no losses due to participation rejection or absence of an ultrasound window, resulting in a total of 96 included patients. Demographic characteristics and ultrasound parameters are shown in Table 1.

The double-check protocol identified an alternative diagnosis in 32 of the included patients (33.3%) (Table 2), leading to therapeutic and/or clinical strategy changes (change of location, admission/discharge decision) in all of them. Among the 64 patients in whom the protocol reaffirmed the diagnosis of AHF, a clinically relevant echocardiographic finding was present in 47 (73.4%), including 17 (26.6%) with previously unknown LVEF < 40% and 42 (65.6%) with previously unknown or undescribed moderate or severe mitral, aortic, or tricuspid valve disease. Overall, the protocol’s application had an impact on 47% of all included patients, either through the identification of an alternative diagnosis or the discovery of additional/relevant pathology in patients with confirmed AHF diagnosis.

**Fig. 1:**
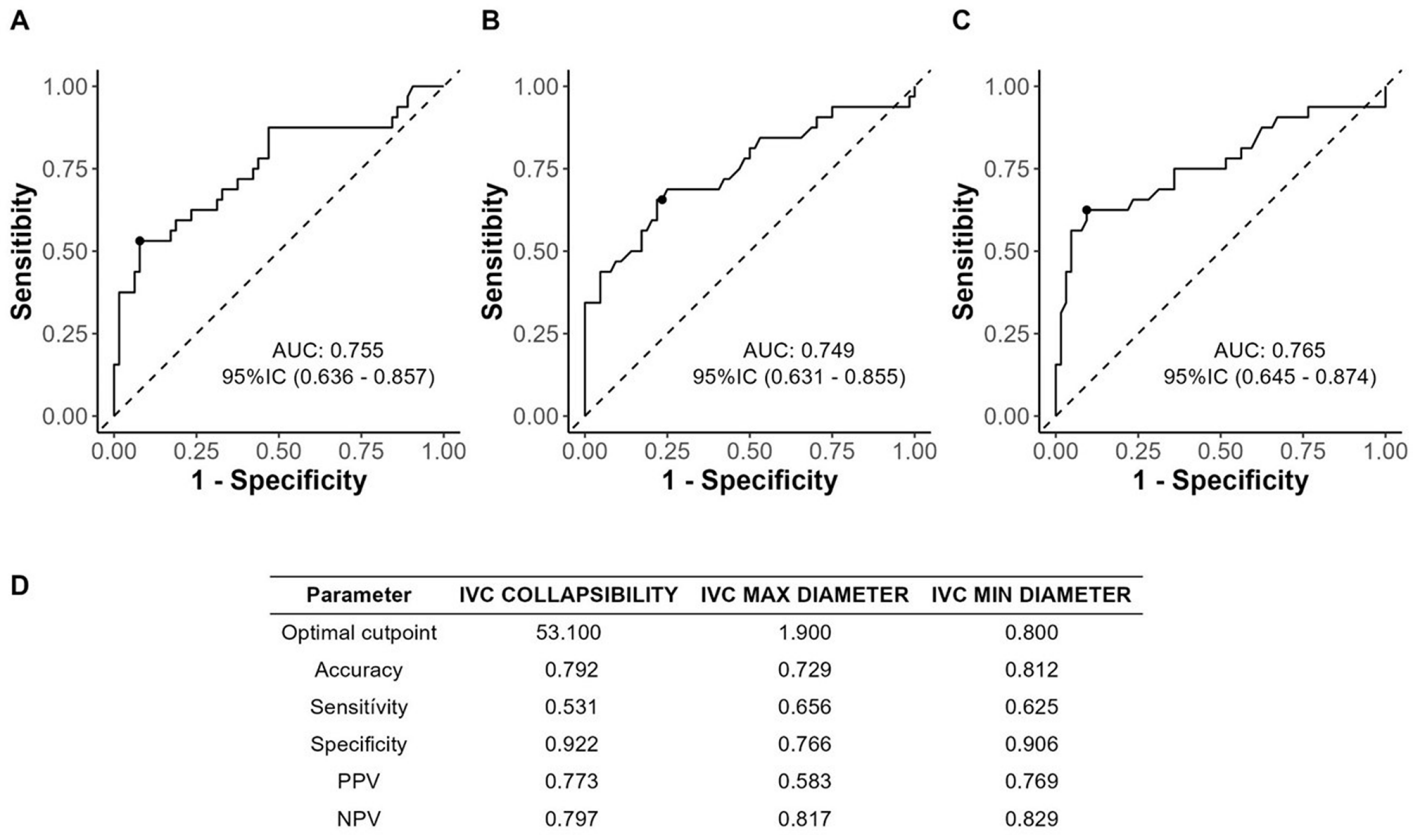
Diagnostic accuracy values for acute heart failure (AHF) of the inferior vena cava (maximum and minimum diameters, collapsibility index)

Analyzing each ultrasound examination separately, the accuracy of the inferior vena cava evaluation is shown in Fig. 1.

**Fig. 2:**
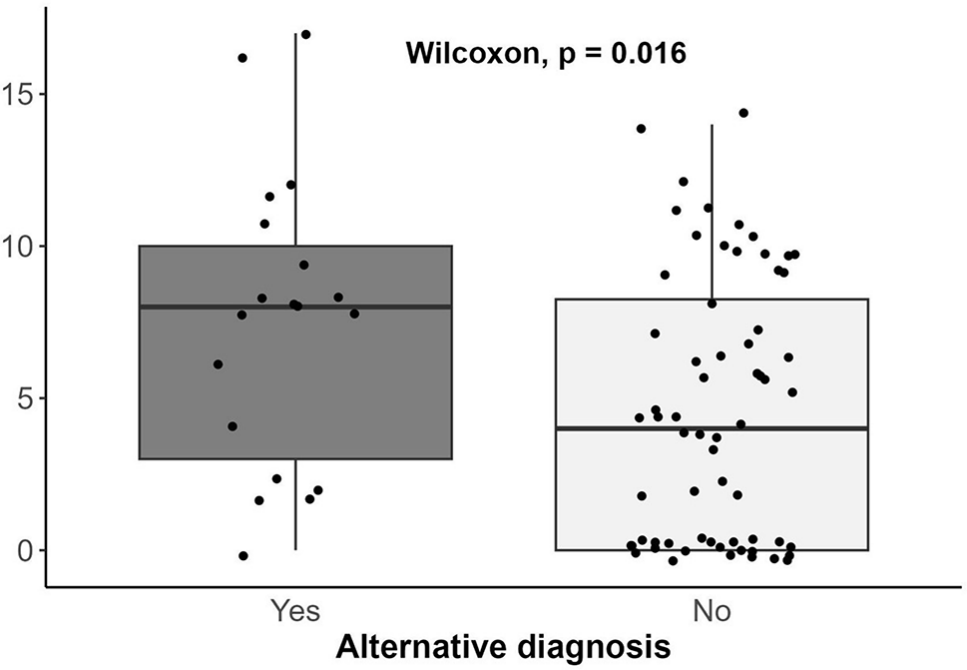
Box plot displaying the asymmetry index

Regarding the lung pattern, the difference in the lung score was calculated not compared to all patients with an alternative diagnosis, but to patients diagnosed with pulmonary pathology (PE, respiratory infection, and pneumonia). As distinguishing a B-pattern between cardiogenic cause and others involves symmetry, a numerical asymmetry index was created, defined by the absolute value of the B-line difference between equivalent quadrants, with a correction factor involving a multiplication by 2 when there were two equivalent zones with a difference of 0, to accentuate the effect of asymmetry. Thus, the index ranged between 0 (completely symmetric) and 20 (completely asymmetric). The distribution of this lung asymmetry index between patients with and without an alternative diagnosis is shown in Fig. 1. Additionally, to observe the densitometric distribution of B-lines by regions, in addition to the lung score, a density map was created using the median of B-lines globally and adjusted for evolution time, as seen in Fig. 2.

**Fig. 3:**
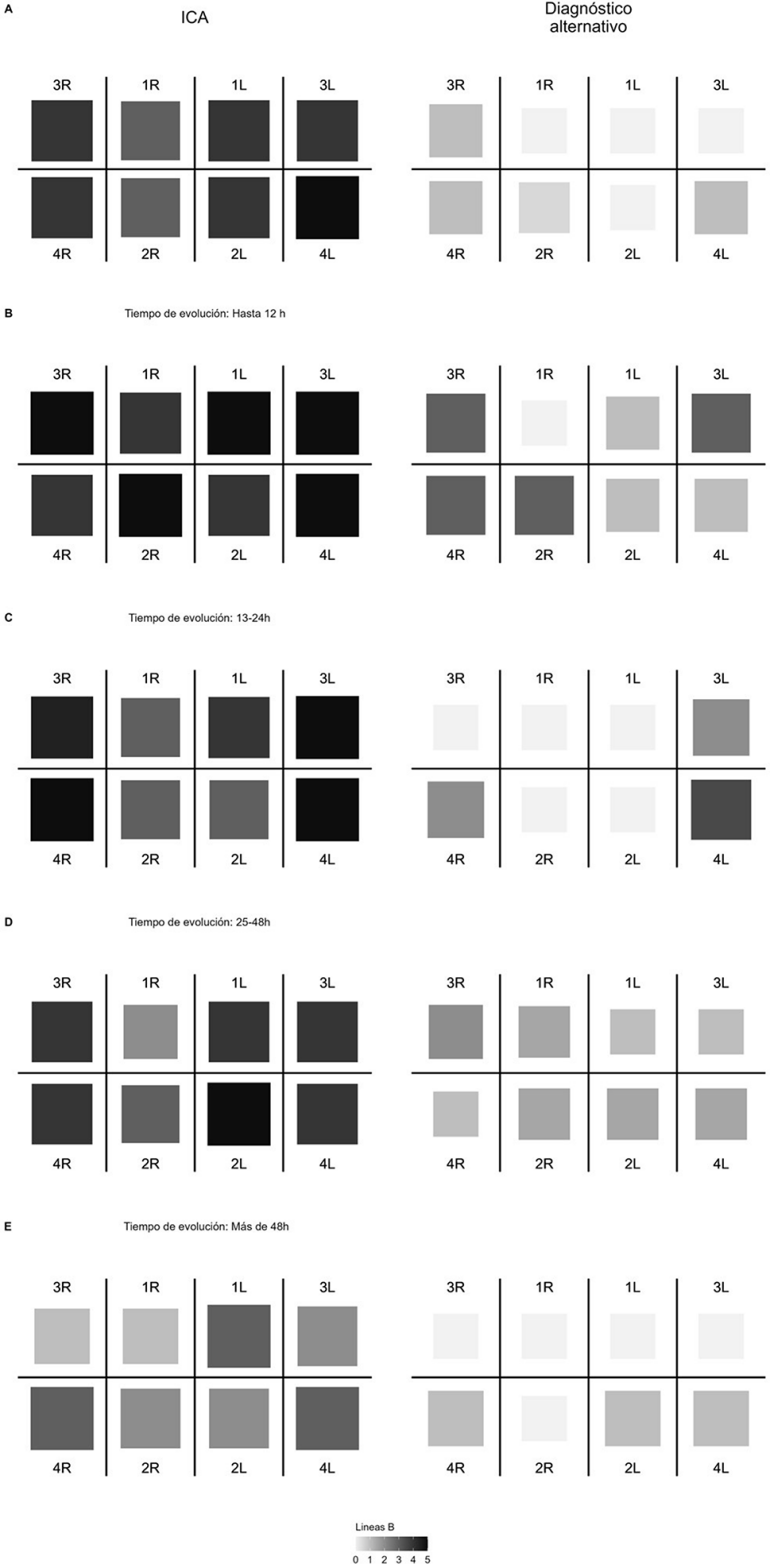
Map of median B-lines density by quadrant, basal (A), and according to time of evolution

With all variables included, a decision tree was constructed using the variables that our statistical model suggested were of greater significance in establishing a strategy for detecting an alternative diagnosis, shown in Fig. 3.

Regarding “particular” alternative diagnosis that could hypothetically be part of the AHF clinical spectrum, in our series, nor pericardial effusions nor the unilateral pleural effusion were associated to AHF in the final discharge medical report. In addition, the patient diagnosed as hypertensive emergency after the protocol application was discharged from the ED without diuretics, making the AHF diagnosis, at least, unlikely.

## Discussion

In this study, we highlight the importance of adding POCUS evaluation to patients diagnosed with AHF in the ED. The proportion of misclassified patients is consistent with available literature [9] and presents a particularly concerning number, mainly because initiating specific AHF treatment can be detrimental for conditions detected as alternatives. Notably, the high number of significant pericardial effusions detected draws attention. This phenomenon was previously described by Blaivas [10], where misclassification as “classic AHF” and initiation of intensive diuretic treatment could potentially lead to hemodynamic instability.

This study also introduces two concepts not previously described in the literature: the asymmetry index and the temporal progression of B-lines adjusted for evolution time. The asymmetry index quantifies differences between interstitial pneumonia caused by SARS-CoV-2 and AHF. Since the distinguishing factor between them is the presence of a symmetric B-pattern [11], we sought to quantify symmetry, leading to the asymmetry index which, with significant implications, opens the potential for application in other patient groups. The index could be valuable in the differential diagnosis between asymmetric interstitial pathologies (interstitial pneumonia, ARDS) and symmetric interstitial or alveolar pathologies (pulmonary fibrosis or AHF), given that both circumstances exhibit a bilateral B-pattern, making B-lines indistinguishable. The second concept, the density of B-lines, offers relevant information, particularly when considering evolution time and the probable location of B-lines. Additionally, it indicates lung reaeration patterns with a centrifugal tendency, as observed in the graphs.

**Fig. 4:**
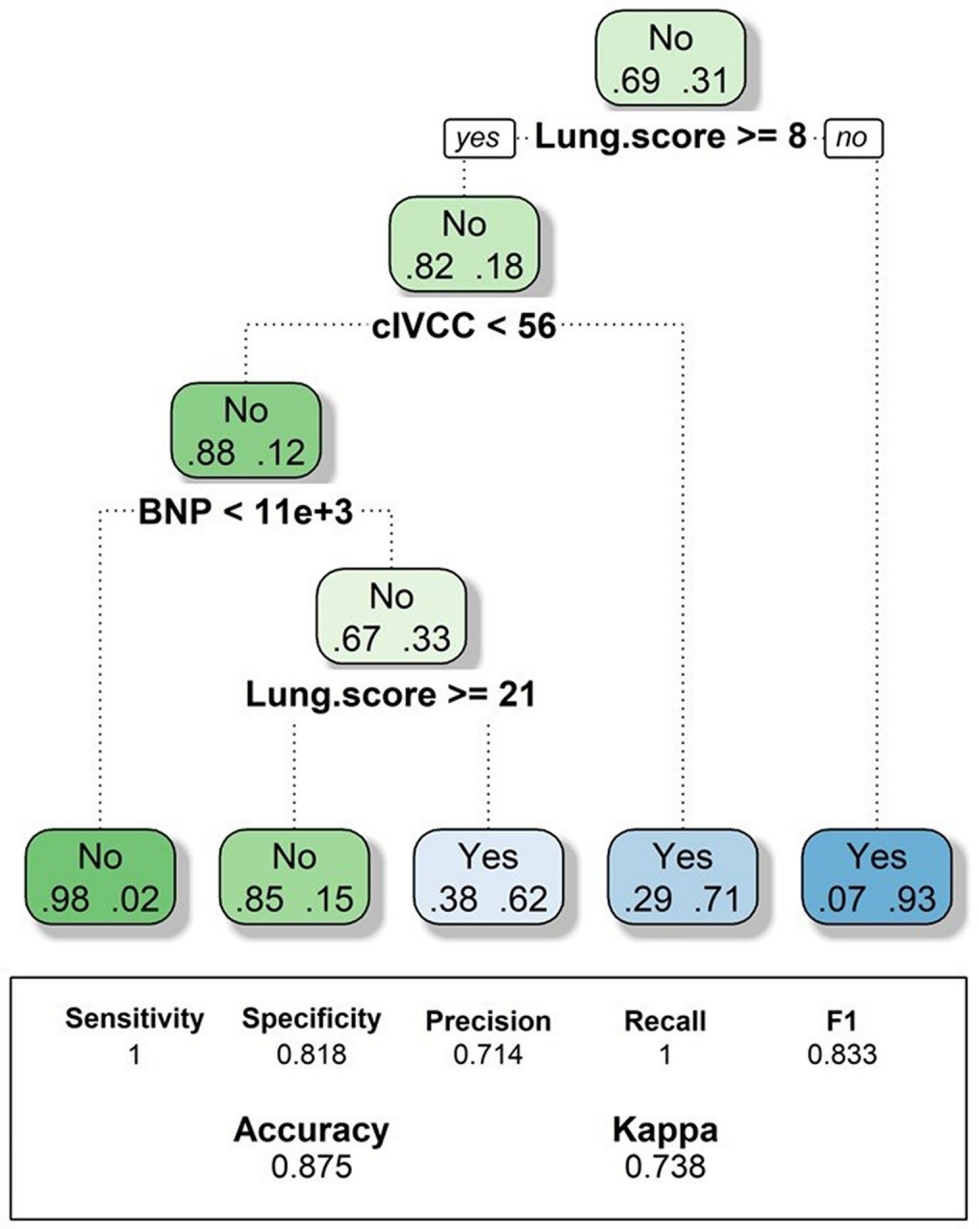
Final decision tree for alternative diagnosis (calculated with a random 80% of sample and tested on remaining 20%)

Regarding the evaluation of the inferior vena cava, the standalone precision values are consistent with other studies in the available literature, but we know that the precision is reinforced with multi-window evaluation, which forms the basis of the decision tree (Fig. 4).

With all these aspects considered, the decision tree suggested by our statistical model exhibits excellent classification ability. It’s worth noting that this model does not consider the presence of significant pericardial effusion initially. Clinically, the initial cardiac assessment appears necessary to exclude both pericardial effusion and acute cor pulmonale patterns, which if absent, would lead to subsequent pulmonary and inferior vena cava evaluations, as suggested. However, we are currently developing a “random forest” model that includes all variables, where both the asymmetry index and the presence of pericardial effusion are incorporated. Initial testing indicates perfect classification ability (100% sensitivity and specificity), though further studies are needed to confirm this.

Conceptually, the study highlights that the competencies required to find an alternative diagnosis don’t demand high expertise and should be part of basic ultrasound training for Emergency Medicine specialists [12]. This is where the study has significance in the day-to-day operations of an Emergency Department. On the other hand, in cases where an alternative diagnosis isn’t found, detecting relevant information for AHF management, such as valve pathology or non-preserved LVEF, requires quantitative measurements (Doppler quantification, biplane Simpson method, etc.) and, thus, more extensive training [13].

Limitations of this study include its single-center nature, conducted in a Level 3 hospital with a high-demand Emergency Department, potentially affecting external validity when applied to centers of different complexity or lower demand. Additionally, patients were already selected since they had been diagnosed with AHF by at least one treating team, rendering the protocol’s application invalid for patients with isolated clinical suspicion of AHF upon ED arrival. Nonetheless, the information derived from the early-stage B-line density map along with the asymmetry index could be applicable in these cases. Moreover, all diagnostic precision values need contextualization due to the highly selected patient population and the prevalence environment in which they are found. While the initial sample size calculation suggested around 87 patients for the main variable (detection of an alternative diagnosis), a larger sample size might be advisable to strengthen the description of the other variables or to include a larger cohort to test the protocol’s application. Finally, given the baseline characteristics of the patients included in this study always considering the main diagnosis, the possibility of the temporal coincidence of a respiratory infection and heart failure is not reflected, something that, according to some studies, is relatively common [14] an can be consider as a main limitation.

## Conclusion

In a population of patients diagnosed with AHF in an Emergency Department, the application of POCUS in the form of a established double-check protocol aids in identifying misclassified patients and provides additional relevant clinical information for the remaining patients where AHF is confirmed. Furthermore, this study introduces novel statistically significant tools for evaluating various lung B-patterns, opening up possibilities for their utilization.

**Table 1:** Demographics and ultrasound parameters

	Overall, *N* = 96^1^	Alternative diagnosis		
		**No**, *N* = 64^1^	**Yes**, *N* = 32^1^	p-value^2^
Age	83 [73–88]	83 [71–89]	84 [77–87]	0.533
Sex				0.428
Male	44 (46.3%)	31 (49.2%)	13 (40.6%)	
Female	51 (53.7%)	32 (50.8%)	19 (59.4%)	
Waiting time in ED (h)	22.0 [14.0-34.5]	21.0 [13.0-27.5]	23.0 [15.8–40.5]	0.490
BNP (pg/ml)	4,658.5 [2,223.8–11,428.3]	5,072.5 [2,304.3-9,110.3]	3,393.5 [1,537.0–13,121.0]	0.649
IVC basal diameter (max)	2.03 ± 0.50	2.16 ± 0.41	1.77 ± 0.55	**< 0.001**
IVC inspiratory diameter (min)	1.43 ± 0.64	1.62 ± 0.51	1.04 ± 0.70	**< 0.001**
IVC Collapsibility index	25.91 [17.13–48.92]	20.86 [15.19–33.01]	53.16 [24.67–59.39]	**< 0.001**
Lung score	23.5 [10.8–31.3]	27.0 [20.8–34.0]	7.5 [4.0-16.8]	**< 0.001**
Diastolic dysfunction type				**< 0.001**
1	12 (13.3%)	5 (7.9%)	7 (25.9%)	
2	9 (10.0%)	2 (3.2%)	7 (25.9%)	
3	21 (23.3%)	15 (23.8%)	6 (22.2%)	
4	9 (10.0%)	9 (14.3%)	0 (0.0%)	
Non-valuable (AFib)	39 (43.3%)	32 (50.8%)	7 (25.9%)	

^1^Mean ± SD; Median [IQR]; n (%)

^2^Wilcoxon rank sum test; Pearson’s Chi-squared test; Fisher’s exact test

**Table 2:** Alternative diagnosis detected by the protocol

	*N* = 32
Respiratory infection (no PNA)	10 (31.2%)
Pneumonia	10 (31.2%)
Pericardial effusion	9 (28.1%)
Pulmonary embolism	1 (3.1%)
Unilateral pleural effusion (no HF)	1 (3.1%)
HTN emergency (no HF)	1 (3.1%)

## Data Availability

Availability of data and supplemental material can be requested if needed.
